# Associação entre Bloqueio Atrioventricular e Mortalidade em Pacientes de Atenção Primária: O Estudo CODE

**DOI:** 10.36660/abc.20210763

**Published:** 2022-07-13

**Authors:** Gabriela Miana de Mattos Paixão, Emilly M. Lima, André B. Quadros, Daniel P. R. Cabral, Renato R. Coelho, Derick M. Oliveira, Jamil de Souza Nascimento, Paulo R. Gomes, Antonio L. Ribeiro

**Affiliations:** 1 Universidade Federal de Minas Gerais Belo Horizonte MG Brasil Universidade Federal de Minas Gerais, Belo Horizonte, MG – Brasil; 2 Faculdade da Saúde e Ecologia Humana Vespasiano MG Brasil Faculdade da Saúde e Ecologia Humana, Vespasiano, MG – Brasil; 3 Ministério da Saúde Superintendência Estadual do Ministério da Saúde em Minas Gerais Belo Horizonte MG Brasil Ministério da Saúde, Superintendência Estadual do Ministério da Saúde em Minas Gerais (SEMS-MG), Belo Horizonte, MG – Brasil

**Keywords:** Doenças Cardiovasculares/complicações, Bloqueio Atrioventricular/fisiopatologia, Bloqueio Atrioventricular/complicações, Mortalidade, Diagnóstico por Imagem, Eletrocardiografia/métodos

## Abstract

**Fundamento:**

O bloqueio atrioventricular (BAV) descreve um comprometimento na condução dos átrios para os ventrículos. Embora o curso clínico do BAV tenha sido avaliado, os achados são de países de alta renda e, portanto, não podem ser extrapolados para a população latina.

**Objetivo:**

Avaliar a associação entre BAV e mortalidade.

**Métodos:**

Foram incluídos pacientes do estudo CODE (Clinical Outcomes in Digital Electrocardiology), maiores de 16 anos que realizaram eletrocardiograma (ECG) digital de 2010 a 2017. Os ECGs foram relatados por cardiologistas e por software automatizado. Para avaliar a relação entre BAV e mortalidade, foram utilizados o modelo log-normal e as curvas de Kaplan-Meier com valores de p bicaudais < 0,05 considerados estatisticamente significativos.

**Resultados:**

O estudo incluiu 1.557.901 pacientes; 40,23% eram homens e a média de idade foi de 51,7 (DP ± 17,6) anos. Durante um seguimento médio de 3,7 anos, a mortalidade foi de 3,35%. A prevalência de BAV foi de 1,38% (21.538). Os pacientes com BAV de primeiro, segundo e terceiro graus foram associados a uma taxa de sobrevida 24% (taxa de sobrevida relativa [RS] = 0,76; intervalo de confiança [IC] de 95%: 0,71 a 0,81; p < 0,001), 55% (RS = 0,45; IC de 95%: 0,27 a 0,77; p = 0,01) e 64% (RS = 0,36; IC de 95%: 0,26 a 0,49; p < 0,001) menor quando comparados ao grupo controle, respectivamente. Os pacientes com BAV 2:1 tiveram 79% (RS = 0,21; IC de 95%: 0,08 a 0,52; p = 0,005) menor taxa de sobrevida do que o grupo controle. Apenas Mobitz tipo I não foi associado a maior mortalidade (p = 0,27).

**Conclusão:**

BAV foi um fator de risco independente para mortalidade geral, com exceção do BAV Mobitz tipo I.

## Introdução

O nó atrioventricular (AV) é responsável pela conexão elétrica entre os átrios e os ventrículos.^1^ A presença de atraso ou interrupção na condução AV é denominada bloqueio atrioventricular (BAV),^2^ que é classificado em três graus, de acordo com a apresentação do eletrocardiograma (ECG).^3^ As causas conhecidas do BAV são várias, incluindo cardiopatia isquêmica, doença degenerativa do sistema de condução, cardiopatia congênita, doença do tecido conjuntivo, doenças inflamatórias, medicamentos e aumento do tônus autonômico.^4^

A prevalência do BAV varia entre 0,6% a 6,04% na literatura, dependendo da população estudada e do grau de BAV.^5 , 6^ A prevalência é geralmente maior em idosos e em homens.^5^ O BAV de primeiro grau é o mais comum e pode ser encontrado com frequência em pacientes com desfechos.^4^

O curso clínico do BAV de primeiro grau tem sido avaliado em estudos com amostras de base comunitária, como a coorte de Framingham.^4^ Pacientes com BAV de primeiro grau têm maior risco de fibrilação atrial,^7^ morte, acidente vascular cerebral ou hospitalização por insuficiência cardíaca.^8^ Também é descrito que, em pacientes com infarto agudo do miocárdio, o BAV de alto grau está associado a um risco aumentado de morbimortalidade.^9^

No entanto, não há estudo prospectivo sobre o valor prognóstico de todos os graus de BAV em uma população geral, o que limita a compreensão do significado dessas anormalidades no ambiente ambulatorial. De fato, estudos anteriores do nosso grupo mostraram que anormalidades do ECG consideradas prognosticamente importantes, como a síndrome de pré-excitação, não têm impacto prognóstico em um ambiente comunitário.^10^ Em contraste, o risco de mortalidade para um paciente com bloqueio de ramo direito é quase tão alto quanto o de um paciente com bloqueio de ramo esquerdo,^11^ embora este último seja considerado um marcador de risco muito mais forte na prática cardiológica geral. O estudo CODE (Clinical Outcomes in Digital Electrocardiology) é um grande banco de dados que compreende todos os ECGs realizados majoritariamente em unidades básicas de saúde pela Rede Telessaúde de Minas Gerais, Brasil, no período de 2010 a 2017.^12^ O banco de dados de ECG foi vinculado ao Sistema de Informação sobre Mortalidade nacional e pode fornecer informações epidemiológicas em uma população que é representativa da população geral. Assim, no presente estudo, objetivamos descrever a prevalência e os fatores de risco do BAV e, principalmente, avaliar a associação entre o BAV e a mortalidade geral nesta grande coorte brasileira de atenção primária.

## Métodos

### Delineamento do estudo

Foi realizado um estudo retrospectivo utilizando um banco de dados de ECGs digitais da Rede Telessaúde de Minas Gerais.^13^ Foi analisado o conjunto de dados do estudo CODE,^12 , 14^ que compreende todos os ECGs válidos realizados em pacientes maiores de 16 anos pela Rede Telessaúde de Minas Gerais de 2010 a 2017. Foram excluídos exames sem traçados válidos ou com problemas técnicos. Nos pacientes que realizaram mais de um ECG, foi analisado apenas o primeiro exame.

### Coleta de dados

Os dados clínicos foram coletados por meio de um questionário padronizado, que incluiu idade, sexo e comorbidades autorreferidas, tais como: hipertensão, diabetes, tabagismo, doença de Chagas, infarto prévio do miocárdio e doença pulmonar obstrutiva crônica.

Os ECGs foram realizados pelo profissional da atenção primária local, utilizando eletrocardiógrafos digitais da Tecnologia Eletrônica Brasileira, modelo ECGPC (São Paulo, Brasil) ou Micromed Biotecnologia, modelo ErgoPC (Brasília, Brasil).

Um software específico, desenvolvido internamente, foi capaz de capturar o traçado do ECG, fazer o upload do ECG e do histórico clínico do paciente e, então, enviá-los para o centro de análise da Rede Telessaúde de Minas Gerais pela internet. As informações clínicas, traçados de ECG e relatórios foram armazenados em um banco de dados específico. Os laudos de ECG foram gerados em modelo de texto livre por cardiologistas e, também, interpretados e codificados automaticamente em códigos de Glasgow e Minnesota pelo programa de análise de ECG de Glasgow 12 derivações (versão 28.4.1, emitido em 16 de junho de 2009).^15^

### Definição de bloqueio atrioventricular

Os laudos médicos foram realizados por uma equipe de 14 cardiologistas treinados, utilizando critérios padronizados. Cada ECG foi interpretado por apenas um cardiologista. O diagnóstico eletrocardiográfico de BAV foi dividido em: BAV de primeiro grau, BAV de segundo grau Mobitz tipo I, BAV de segundo grau Mobitz tipo II, BAV 2:1, BAV de alto grau e BAV de terceiro grau^3^ ( Tabela 1 ). No presente estudo, não incluímos Mobitz tipo II devido à baixa prevalência (7 casos) e BAV de alto grau (6 casos) foi agrupado em BAV de terceiro grau para análise.

**Tabela 1:** Definição e classificação de bloqueio atrioventricular3

Tipo de BAV	Definição
Primeiro grau	Ondas P associadas à condução atrioventricular 1:1 e intervalo PR > 200 ms
Segundo grau Mobitz tipo I	Ondas P com frequência constante (< 100 bpm) com uma única onda P periódica não conduzida associada a ondas P antes e depois da onda P não conduzida, com intervalos PR inconstantes
Segundo grau Mobitz tipo II	Ondas P com frequência constante (< 100 bpm) com uma única onda P periódica não conduzida associada a outras ondas P antes e depois da onda P não conduzida com intervalos PR constantes (excluindo bloqueio atrioventricular 2:1)
2:1	Ondas P com uma frequência constante (ou quase constante devido à arritmia sinusal ventriculofásica) (< 100 bpm) onde 1 em cada 2 ondas P conduz para os ventrículos
Alto grau	≥ 2 ondas P consecutivas a uma taxa fisiológica constante que não conduzem para os ventrículos, com evidência de alguma condução atrioventricular
Terceiro grau	Nenhuma evidência de condução atrioventricular

*BAV: bloqueio atrioventricular.*

Os laudos de ECG foram gerados como um texto livre não organizado. Para reconhecer o diagnóstico de BAV entre mais de um milhão de relatórios, foi usado o aprendizado de máquina hierárquico de texto livre. Primeiramente, o texto foi pré-processado removendo palavras de parada e gerando n-gramas. Em seguida, utilizou-se o modelo de classificação denominado *Lazy Associative Classifier,* ^16^ que foi construído com um dicionário de 2.800 amostras criado manualmente por especialistas com base em textos de diagnósticos reais. O relatório final foi obtido imputando os resultados da *Lazy Associative Classifier* a uma árvore de decisão para desambiguação de classes. A árvore de decisão foi treinada usando o conjunto original de dados. O modelo de classificação foi testado em 4.557 laudos médicos rotulados manualmente por 2 cardiologistas com 99% de acurácia, 100% de valor preditivo positivo e 99% de sensibilidade.^17^

**Figura 1:**
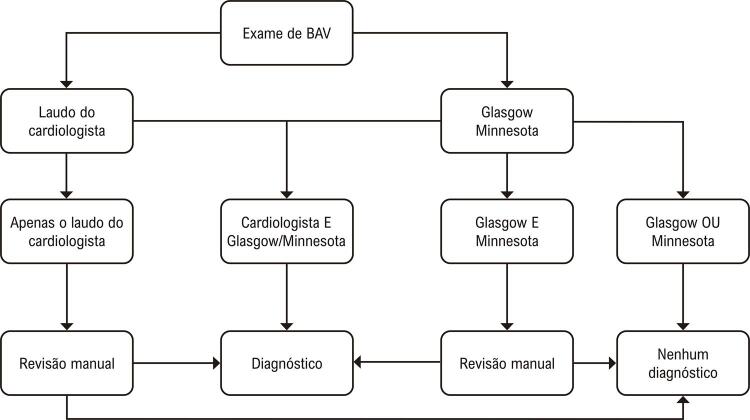
Diagrama para diagnóstico de bloqueio atrioventricular no banco de dados de ECG. BAV: bloqueio atrioventricular.

O diagnóstico eletrocardiográfico de BAV foi considerado automaticamente quando houve concordância entre o laudo do cardiologista e o laudo automático no código de Glasgow ou Minnesota. Nos casos em que houve discordâncias entre o laudo médico e um dos programas automáticos, foi realizada uma revisão manual de 9.038 ECGs por uma equipe treinada. Não foram considerados os casos em que o BAV foi diagnosticado por apenas um dos sistemas automáticos ( Figura 1 ). O grupo controle foi composto por pacientes sem nenhum tipo de BAV.

### Ligação probabilística

A coorte eletrônica foi obtida ligando os dados dos exames de ECG (nome, sexo, data de nascimento, cidade de residência) e os do Sistema de Informação sobre Mortalidade nacional,^12^ utilizando métodos padrão de ligação probabilística (FRIL: *fine-grained record linkage* software, v. 2.1.5, Atlanta, GA).^12 , 18^

### Análise estatística

O programa R (versão 3.4.3, Viena, Áustria) foi utilizado para análise estatística. Os dados categóricos foram relatados como contagens e porcentagens; as variáveis contínuas foram relatadas como média e desvio padrão (DP). O desfecho foi mortalidade por todas as causas, incluindo todos os códigos da Classificação Internacional de Doenças na certificação médica da causa de óbito. Foi utilizado o teste de Shapiro-Wilk para verificar a normalidade dos dados. O método de Kaplan-Meier foi utilizado para estimar as curvas de sobrevida para todas as causas de óbito. Utilizamos o teste da razão de verossimilhança (TRV) para ajustar os dados para o melhor modelo paramétrico, uma vez que a suposição proporcional para o modelo de regressão de Cox foi violada. No TRV, o modelo generalizado, representado pelo modelo de regressão gamma generalizado, foi comparado com os demais modelos de interesse (Weibull e log-normal). Optamos por trabalhar com o modelo log-normal, pois a log-probabilidade desse modelo era maior e a análise de resíduos indicou que a distribuição log-normal foi a melhor escolha para esses dados. A taxa de sobrevida relativa (SR) foi utilizada como medida de associação, com intervalo de confiança de 95%. SR < 1 significa maior risco de mortalidade e SR > 1 significa risco menor. Valores de p bicaudais < 0,05 foram considerados estatisticamente significativos. Este estudo foi aprovado pelo Comitê de Ética em Pesquisa da Universidade Federal de Minas Gerais.

## Resultados

Em total, foram incluídos 1.557.901 pacientes; 40,23% eram homens e a média de idade foi de 51,67 (DP ± 17,58) anos. Durante um seguimento médio de 3,7 anos, a mortalidade foi de 3,35%. A prevalência de BAV foi de 1,38% (21.538); 1,32% (20.644) correspondendo ao BAV de primeiro grau, 0,02% (273) ao BAV de segundo grau e 0,04% (621) ao BAV de terceiro grau. Dentre esses, 273 casos eram de BAV de segundo grau, 212 eram Mobitz tipo I e 61 eram 2:1. As condições clínicas de todos os pacientes estão descritas na Tabela 2 .

**Tabela 2:** Dados basais dos pacientes, de acordo com a presença de bloqueio atrioventricular e respectivo grau

	Sem BAV n = 1.536.363	BAV de primeiro grau n = 20.644	OR* ajustado	BAV de segundo grau n = 273	OR* ajustado	BAV de terceiro grau n = 621	OR* ajustado
Idade (anos)	51,5 (17,5)	64,9 (16,9)	-	61,7 (19,8)	-	66,6 (17,5)	-
Sexo masculino	615.097 (40)	11.176 (54,1)	-	164 (60,1)	-	286 (46,1)	-
Hipertensão	492.488 (32,1)	9370 (45,4)	1,23 (1,19-1,26)	100 (36,6)	0,89 (0,69-1,15)	298 (48,0)	1,18 (1,01-1,39)
Diabetes	100.844 (6,6)	1826 (8,8)	1,10 (1,05-1,15)	18 (6,6)	0,87 (0,52-1,36)	55 (8,9)	1,05 (0,78-1,37)
Tabagismo atual	107.346 (7,0)	1384 (6,7)	0,90 (0,85-0,95)	20 (7,3)	0,93 (0,57-1,43)	51 (8,2)	1,21 (0,90-1,60)
Doença de Chagas	33.134 (2,2)	1336 (6,5)	2,76 (2,60-2,92)	35 (12,8)	6,04 (4,16-8,50)	81 (13,0)	5,75 (4,52-7,23)
Infarto do miocárdio	11.286 (0,7)	304 (1,5)	1,48 (1,31-1,66)	0 (0,0)	-	11 (1,8)	1,80 (0,93-3,10)
DPOC	11.029 (0,7)	231 (1,1)	1,14 (1,00-1,30)	0 (0,0)	-	4 (0,6)	0,64 (0,20-1,49)

Os dados são apresentados como média (desvio padrão) ou número (%). BAV: bloqueio atrioventricular; DPOC: doença pulmonar obstrutiva crônica; OR: odds ratio. *Idade, sexo, hipertensão, diabetes, tabagismo atual, doença de Chagas e doença pulmonar obstrutiva crônica.

**Figura 2:**
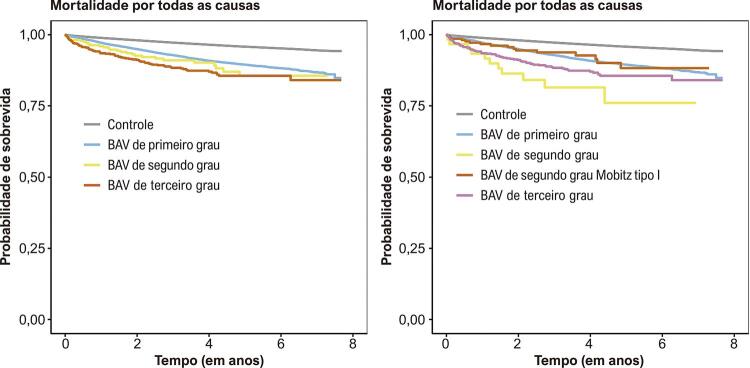
Curvas de sobrevida de Kaplan-Meier, de acordo com o subtipo de bloqueio atrioventricular. BAV: bloqueio atrioventricular.

Após ajuste para sexo, idade e condições clínicas, os pacientes com BAV de primeiro, segundo e terceiro graus foram associados a uma taxa de sobrevida 24%, 55% e 64% menor quando comparado ao grupo controle, respectivamente ( Figura 2 ). Na análise de sobrevida dividida por subtipo de BAV, apenas o segundo grau Mobitz tipo I não foi associado a maior mortalidade. Pacientes com BAV 2:1 tiveram taxa de sobrevida 79% menor do que o grupo controle, enquanto os com BAV de terceiro grau tiveram 64% ( Tabela 3 ; Figura 2 ).

**Tabela 3:** Valor prognóstico dos pacientes com subtipos de bloqueio atrioventricular

	SR (IC de 95%)		
Tipo de BAV	Modelo 1: Não ajustado	Modelo 2: Ajustado para idade e sexo	Modelo 3: Ajustado para variáveis clínicas*
Primeiro grau	0,24 (0,23-0,26)	0,73 (0,69-0,78)	0,76 (0,71-0,81)
Mobitz I	0,26 (0,13-0,50)	0,63 (0,33-1,20)	0,65 (0,34-1,24)
2:1	0,05 (0,02-0,13)	0,20 (0,08-0,50)	0,21 (0,09-0,52)
Terceiro grau	0,11 (0,08-0,15)	0,34 (0,25-0,46)	0,36 (0,26-0,49)

BAV: bloqueio atrioventricular; IC: intervalo de confiança; SR: taxa de sobrevida relativa. *Idade, sexo, hipertensão, diabetes, tabagismo atual, doença de Chagas e doença pulmonar obstrutiva crônica.

## Discussão

Nesta grande coorte eletrônica com mais de um milhão de pacientes, o BAV foi associado a maior risco de mortalidade geral. Em relação ao tipo de BAV, apenas o Mobitz tipo I não apresentou risco aumentado de mortalidade, comparado ao grupo controle.

Em pacientes com cardiopatia estrutural, o BAV de primeiro grau tem sido descrito como um fator de risco para desfechos adversos.^19 , 20^ Por outro lado, estudos longitudinais anteriores na população geral, que incluíram principalmente homens jovens e de meia-idade, verificaram que o intervalo PR prolongado tem um curso benigno.^21 - 23^ Devemos destacar que esses dados vieram de uma população específica com vigilância limitada e uma amostra relativamente baixa de pacientes com BAV. Mais recentemente, uma publicação da coorte de Framingham^4^ mudou esse paradigma. Após 20 anos de seguimento, o prolongamento do PR foi associado ao aumento do risco de fibrilação atrial, implante de marcapasso e óbito^4^ . Um grande estudo de ECG dinamarquês com 288.181 pacientes confirmou o maior risco de fibrilação atrial associado à presença do primeiro BAV.^24^

Em nossa população, foi encontrada uma redução de 24% na sobrevida de pacientes com PR > 200 ms, após ajuste para idade, sexo e condições clínicas, em contraste com um estudo anterior na população finlandesa.^25^ Devem ser apontadas algumas diferenças entre essas coortes. A coorte brasileira era mais velha (média de idade 51,7 versus 44 anos) e também incluia pacientes idosos. Analisamos cerca de 1,5 milhão de ECG contra 10 mil. A doença de Chagas foi relativamente prevalente e teve forte associação com a presença de BAV, independente do grau. As diferenças sociais entre os dois países também podem ter contribuído. O acesso aos serviços públicos de saúde e a educação da população são completamente desiguais em países de baixa e média renda e podem ter impacto prognóstico na população.^26^

Está bem estabelecido que BAV Mobitz tipo II irreversível, BAV de alto grau e BAV de terceiro grau são indicações para estimulação permanente, mesmo em pacientes assintomáticos.^3^ Sua associação com a mortalidade é esperada,^9^ pois a lesão da condução AV é mais grave e a cardiopatia está frequentemente relacionada.^3^ O prognóstico no BAV 2:1 está intimamente relacionado ao local do BAV: nodal ou infranodal.^3^ No presente estudo, o BAV 2:1 no ECG de 12 derivações foi associado a uma redução de 79% na sobrevida relativa, provavelmente indicando um bloqueio infranodal. O BAV Mobitz tipo I, por outro lado, não foi associado a maior mortalidade em nossa coorte.

BAV Mobitz tipo I frequentemente tem prognóstico benigno, principalmente em pacientes jovens sem doença cardíaca.^27^ Pode ser um BAV mediado vagal que não apresenta comprometimento anatômico da condução AV,^28^ não evoluindo, portanto, para BAV de alto grau. Em pacientes mais velhos, a história natural pode ser diferente, e eles podem se beneficiar de um marcapasso permanente.^29^ Não realizamos subanálise em pacientes idosos e a presença de sintomas é desconhecida.

Os pacientes com emergências cardiovasculares frequentemente procuram atendimento em unidades básicas de saúde, principalmente em municípios pequenos e remotos. Os serviços de tele-eletrocardiografia desempenham um papel importante nesse cenário, principalmente por reconhecerem anormalidades de ECG potencialmente fatais que são diagnosticadas erroneamente pelo médico local.^30^ Em nosso serviço, o BAV de segundo grau foi estatisticamente maior nos ECGs assinalados como eletivos do que naqueles com prioridade de emergência^30^ . Os desfechos dos pacientes podem mudar com o encaminhamento precoce ao hospital e consequente implante de marcapasso.^31^ Os dados de hospitalização não estavam disponíveis para toda a nossa coorte e, portanto, não foram incluídos no presente artigo. No entanto, futuros trabalhos estão planejados nesta área para avaliar a jornada dos pacientes em nosso sistema de saúde a partir do diagnóstico de BAV por ECG.

### Limitações

O nosso estudo possui algumas limitações. Os dados clínicos foram autorrelatados e, portanto, podem ter sido subnotificados. O software *Lazy Associative Classifier* usado para classificar laudos de ECG tem boa precisão, sensibilidade e valor preditivo positivo, mas pode cometer erros. Para minimizar esse problema, incluímos a classificação automática de Glasgow e Minnesota no algoritmo diagnóstico. Além disso, foi realizada a revisão manual de mais de 9.000 ECGs para confirmar a presença de BAV. A ligação probabilística também apresenta alguns problemas, tais como sensibilidade menos que perfeita e a possibilidade de pares falsos. Portanto, definimos um alto ponto de corte para pares verdadeiros e realizamos revisão manual

para os casos duvidosos. Ainda não temos informações sobre sintomas ou dados de internação, mas os dados de procedimentos de marcapasso em cada grupo estarão disponíveis em breve para análise e têm sido planejados trabalhos futuros sobre esse tema.

Apesar disso, nosso estudo traz novos dados sobre o prognóstico do BAV, visto que avalia uma população latina de um centro de atenção primária com mais de um milhão de pacientes. Os nossos achados são consistentes e podem constituir uma ferramenta útil para direcionar políticas de saúde pública e recursos de financiamento.

## Conclusão

A presença de BAV foi associada a um risco aumentado de mortalidade geral na população da Rede Telessaúde de Minas Gerais. Nos pacientes com BAV de segundo e terceiro grau, apenas aqueles com Mobitz tipo I não apresentaram maior risco de mortalidade.
