# Supplementary material for: Associação entre Bloqueio Atrioventricular e Mortalidade em Pacientes de Atenção Primária: O Estudo CODE
Source: Arq Bras Cardiol. 2022 Jul 13;119(4):564–71. [Article in Portuguese] doi: 10.36660/abc.20210763 (PMC9563883; doi:10.36660/abc.20210763)
Supplement: Translation

# Translation: Association between Atrioventricular Block and Mortality in Primary Care Patients: The CODE Study

**Authors:** Gabriela Miana de Mattos Paixão, Emilly M. Lima, André B. Quadros, Daniel P. R. Cabral, Renato R. Coelho, Derick M. Oliveira, Jamil de Souza Nascimento, Paulo R. Gomes, Antonio L. Ribeiro

**Affiliations:** 1 Brazil Universidade Federal de Minas Gerais, Belo Horizonte, MG – Brazil; 2 Brazil Faculdade da Saúde e Ecologia Humana, Vespasiano, MG – Brazil; 3 Brazil Ministério da Saúde, Superintendência Estadual do Ministério da Saúde em Minas Gerais (SEMS-MG), Belo Horizonte, MG – Brazil

**Keywords:** Cardiovascular Diseases/complications, Atrioventricular Block/physiopathology, Atrioventricular Block/complications, Mortality, Diagnostic Imaging, Electrocardiography/methods

## Abstract

**Background:**

Atrioventricular block (AVB) describes an impairment of conduction from the atria to the ventricles. Although the clinical course of AVB has been evaluated, the findings are from high-income countries and, therefore, cannot be extrapolated to the Latinx population.

**Objective:**

Evaluate the association between AVB and mortality.

**Methods:**

Patients from the CODE (Clinical Outcomes in Digital Electrocardiology) study, older than 16 years who underwent digital electrocardiogram (ECG) from 2010 to 2017 were included. ECGs were reported by cardiologists and by automated software. To assess the relationship between AVB and mortality, the log-normal model and the Kaplan-Meier curves were used with two-tailed p-values < 0.05 considered statistically significant.

**Results:**

The study included 1,557,901 patients; 40.2% were men, and mean age was 51.7 (standard deviation ± 17.6) years. In a mean follow-up of 3.7 years, the mortality rate was 3.35%. The AVB prevalence was 1.38% (21,538). Patients with first-, second-, and third-degree AVB were associated with 24% (relative survival rate [RS] = 0.76; 95% confidence interval [CI]: 0.71-0.81; p < 0.001), 55% (RS = 0.45; 95% CI: 0.27-0.77; p = 0.01), and 64% (RS = 0.36; 95% CI: 0.26-0.49; p < 0.001) lower survival rate when compared to the control group, respectively. Patients with 2:1 AVB had 79% (RS = 0.21; 95% CI: 0.08-0.52; p = 0.005) lower survival rate than the control group. Only Mobitz type I was not associated with higher mortality (p = 0.27).

**Conclusion:**

AVB was an independent risk factor for overall mortality, with the exception of Mobitz type I.

## Introduction

The atrioventricular (AV) node is responsible for the electrical connection between the atria and ventricles.^1^ The presence of delay or interruption in AV conduction is called atrioventricular block (AVB),^2^ which is classified into three degrees, according to the electrocardiogram (ECG) presentation.^3^ There are several known causes of AVB, including ischemic heart disease, degenerative conduction system disease, congenital heart disease, connective tissue disease, inflammatory diseases, medications, and increased autonomic tonus.^4^

AVB prevalence varies between 0.6% to 6.04% in the literature, depending on the population studied and the degree of AVB.^5 , 6^ The prevalence is usually higher in the elderly and in men.^5^ First-degree AVB is the most common, and can be frequently found in outcome patients.^4^

The clinical course of first-degree AVB has been evaluated in studies from community-based samples, such as the Framingham cohort.^4^ Patients with first-degree AVB have a higher risk of atrial fibrillation,^7^ death, stroke, or hospitalization for heart failure.^8^ It is also described that, in patients with acute myocardial infarction, high-degree AVB is associated with an increased risk of morbidity and mortality.^9^

Nonetheless, there is no prospective study on the prognostic value of all degrees of AVB in a general population, which limits the understanding of the significance of these abnormalities in an outpatient setting. Indeed, previous studies from our group showed that ECG abnormalities that are considered prognostically important, such as pre-excitation syndrome, have no prognostic impact in a community setting.^10^ In contrast, the risk of mortality for a patient with right bundle branch block (BBB) is almost as high as that of a patient with left BBB,^11^ even though the latter is considered a much stronger marker of risk in general cardiology practice. The CODE (Clinical Outcomes in Digital Electrocardiology) study is a large database that comprises all ECGs performed mostly at primary health care facilities by the Telehealth Network of Minas Gerais, Brazil, from 2010 to 2017.^12^ The ECG database was linked to the Brazilian Mortality Information System and can provide epidemiological information in a population that is representative of the general population. Thus, in the present study, we aim to describe the prevalence and risk factors of AVB and, mainly, to evaluate the association between AVB and overall mortality in this large primary care Brazilian cohort.

## Methods

### Study design

We conducted a retrospective study using a database of digital ECGs from the Telehealth Network of Minas Gerais (TNMG),^13^ The CODE dataset,^12 , 14^ which comprises all valid ECGs performed in patients over 16 years old by the TNMG from 2010 to 2017, was analyzed. Exams without valid tracings or with technical problems were excluded. In patients who underwent more than one ECG, only the first exam was analyzed.

### Data collection

Clinical data were collected using a standardized questionnaire, which included age, sex, and self-reported comorbidities, such as: hypertension, diabetes, smoking, Chagas disease, previous myocardial infarction, and chronic obstructive pulmonary disease.

ECGs were performed by the local primary care professional, using digital electrocardiographs by Tecnologia Eletrônica Brasileira, model ECGPC (São Paulo, Brazil) or Micromed Biotecnologia, model ErgoPC 13 (Brasilia, Brazil).

Specific software, developed in-house, was capable of capturing ECG tracing, uploading the ECG and the patient’s clinical history, and sending them to the TNMG analysis center through the internet. The clinical information, ECG tracings, and reports were stored in a specific database. The ECG reports were generated in a free text model by cardiologists and, also, automatically interpreted and coded into Glasgow and Minnesota codes by the Glasgow 12-lead ECG analysis program (release 28.4.1, issued on June 16, 2009).^15^

### Definition of atrioventricular block

The medical reports were performed by a team of 14 trained cardiologists using standardized criteria. Each ECG was interpreted by only one cardiologist. The electrocardiographic diagnosis of AVB was divided into: first-degree AVB, second-degree Mobitz type I AVB, second-degree Mobitz type II, 2:1 AVB, high-degree AVB, and third-degree AVB^3^ ( Table 1 ). In this study, we did not include Mobitz type II because of the low prevalence (7 cases) and high-degree AVB (6 cases) was grouped into third-degree AVB for the analysis.

**Table 1:** Definition and classification of atrioventricular block3

Type of AVB	Definition
First-degree	P waves associated with 1:1 atrioventricular conduction and a PR interval > 200 ms
Second-degree Mobitz type I	P waves with a constant rate (< 100 bpm) with a periodic single nonconducted P wave associated with P waves before and after the nonconducted P wave with inconstant PR intervals
Second-degree Mobitz type II	P waves with a constant rate (< 100 bpm) with a periodic single nonconducted P wave associated with other P waves before and after the nonconducted P wave with constant PR intervals (excluding 2:1 atrioventricular block)
2:1	P waves with a constant (or near constant rate because of ventriculophasic sinus arrhythmia) rate (< 100 bpm) where every other P wave conducts to the ventricles
High-degree	≥ 2 consecutive P waves at a constant physiologic rate that do not conduct to the ventricles with evidence of some atrioventricular conduction
Third-degree	No evidence of atrioventricular conduction

*AVB: atrioventricular block.*

ECG medical reports were generated as an unorganized free text. In order to recognize AVB diagnosis among more than a million reports, hierarchical free-text machine learning was used. First, the text was preprocessed by removing stop words and generating n-grams. Then, we used the classification model called Lazy Associative Classifier,^16^ which was built with a 2800-sample dictionary manually created by specialists based on text from real diagnoses. The final report was obtained by imputing the Lazy Associative Classifier results to a decision tree for class disambiguation. The decision tree was trained using the original dataset. The classification model was tested on 4557 medical reports manually labeled by 2 cardiologists with 99% accuracy, 100% positive predictive value, and 99% sensibility.^17^

Electrocardiographic diagnosis of AVB was considered automatically when there was agreement between the cardiologist report and the automatic report from Glasgow or Minnesota code. In the cases where there were discordances between the medical report and one of the automatic programs, manual revision of 9038 ECGs was carried out by trained staff. Cases where AVB were diagnosed by only one of the automatic systems were not considered ( Figure 1 ). The control group was composed of patients without any type of AVB.

**Figure 1:** Diagram for atrioventricular block diagnosis in the ECG database. AVB: atrioventricular block.

### Probabilistic linkage

The electronic cohort was obtained linking data from the ECG exams (name, sex, date of birth, city of residence) and those from the Brazilian Mortality Information System,^12^ using standard probabilistic linkage methods (FRIL: fine-grained record linkage software, v.2.1.5, Atlanta, GA).^12 , 18^

### Statistical analysis

R program (version 3.4.3, Vienna, Austria) was used for statistical analysis. Categorical data were reported as counts and percentages; continuous variables were reported as mean and standard deviation (SD). The endpoint was all-cause mortality, including all International Classification of Diseases codes in the medical certification of cause of the death. The Shapiro-Wilk test was used to verify the normality of the data. The Kaplan-Meier method was used to estimate the survival curves for all causes of death. We used the likelihood ratio test (LRT) to adjust data for the best parametric model, since the proportional assumption for the Cox regression model was violated. In the LRT, the generalized model, represented by the generalized gamma regression model, was compared with the other models of interest (Weibull and log-normal). We chose to work with the log-normal model, since the log-likelihood of this model was higher and the residual analysis indicated that log-normal distribution was a better choice for this data. Relative survival rate (RS) was used as the measure of association, with a confidence interval of 95%. RS < 1 means higher risk of mortality, and RS > 1 means lower risk. Two-tailed p-values < 0.05 were considered statistically significant. This study was approved by the Research Ethics Committee of the Federal University of Minas Gerais.

## Results

A total of 1,557,901 patients were included; 40.23% were men, and mean age was 51.67 (SD ± 17.58) years. In a mean follow-up of 3.7 years, the mortality rate was 3.35%. The prevalence of AVB was 1.38% (21,538); 1.32% (20,644) corresponding to first-degree AVB, 0.02% (273) to second-degree AVB, and 0.04% (621) to third-degree AVB. Among these 273 cases of second-degree AVB, 212 were Mobitz type I, and 61 were 2:1. The clinical conditions of all patients are described in Table 2 .

**Table 2:** Dados basais dos pacientes, de acordo com a presença de bloqueio atrioventricular e respectivo grau

	Without AVB n = 1,536,363	First-degree AVB n = 20,644	Adjusted OR*	Second-degree AVB n = 273	Adjusted OR*	Third-degree AVB n = 621	Adjusted OR*
Age (years)	51.5 (17.5)	64.9 (16.9)	-	61.7 (19.8)	-	66.6 (17.5)	-
Male sex	615.097 (40)	11.176 (54.1)	-	164 (60.1)	-	286 (46.1)	-
Hypertension	492.488 (32.1)	9370 (45.4)	1.23 (1.19-1.26)	100 (36.6)	0.89 (0.69-1.15)	298 (48.0)	1.18 (1.01-1.39)
Diabetes	100.844 (6.6)	1826 (8.8)	1.10 (1.05-1.15)	18 (6.6)	0.87 (0.52-1.36)	55 (8.9)	1.05 (0.78-1.37)
Current smoking	107.346 (7.0)	1384 (6.7)	0.90 (0.85-0.95)	20 (7.3)	0.93 (0.57-1.43)	51 (8.2)	1.21 (0.90-1.60)
Chagas disease	33.134 (2.2)	1336 (6.5)	2.76 (2.60-2.92)	35 (12.8)	6.04 (4.16-8.50)	81 (13.0)	5.75 (4.52-7.23)
Myocardial infarction	11.286 (0.7)	304 (1.5)	1.48 (1.31-1.66)	0 (0.0)	-	11 (1.8)	1.80 (0.93-3.10)
COPD	11.029 (0.7)	231 (1.1)	1.14 (1.00-1.30)	0 (0.0)	-	4 (0.6)	0.64 (0.20-1.49)

Data are presented as mean (standard deviation) or number (%). AVB: atrioventricular block; COPD: chronic obstructive pulmonary disease;OR: odds ratio. *Age, sex, hypertension, diabetes, current smoking, Chagas disease, and chronic obstructive pulmonary disease.

After adjustment for sex, age, and clinical conditions, patients with first-, second-, and third-degree AVB were associated with 24%, 55%, and 64% lower survival rate when compared to the control group, respectively ( Figure 2 ). In the survival analysis divided by subtype of AVB, only the second-degree Mobitz type I was not associated with higher mortality. Patients with 2:1 AVB had 79% lower survival rate than the control group, while third-degree AVB had 64% ( Table 3 ; Figure 2 ).

**Figure 2:** Kaplan-Meier survival curves, according to the subtype of atrioventricular block. 1dAVb: first-degree atrioventricular block; 2dAVb: second-degree atrioventricular block; 3dAVb: third-degree atrioventricular block; MI: Mobitz type I.

**Table 3:** Prognostic value of patients with subtypes of atrioventricular block

	RS (95% CI)		
Type of AVB	Model 1: Unadjusted	Model 2: Adjusted for age and sex	Model 3: Adjusted for clinical variables*
First-degree	0,24 (0,23-0,26)	0,73 (0,69-0,78)	0,76 (0,71-0,81)
Mobitz I	0,26 (0,13-0,50)	0,63 (0,33-1,20)	0,65 (0,34-1,24)
2:1	0,05 (0,02-0,13)	0,20 (0,08-0,50)	0,21 (0,09-0,52)
Third-degree	0,11 (0,08-0,15)	0,34 (0,25-0,46)	0,36 (0,26-0,49)

AVB: atrioventricular block; CI: confidence interval; RS: relative survival rate. *Age, sex, hypertension, diabetes, current smoking, Chagas disease and chronic obstructive pulmonary disease.

## Discussion

In this large electronic cohort with more than one million patients, AVB was associated with higher risk of overall mortality. Regarding the type of AVB, only Mobitz type I did not have an increased risk of mortality, compared to the control group.

In patients with structural heart disease, first-degree AVB has been described as a risk factor for adverse outcome.^19 , 20^ On the other hand, previous longitudinal studies in the general population that mainly included young and middle-age men found that prolonged PR interval has a benign course.^21 - 23^ We should highlight that this data came from a specific population with limited surveillance and a relatively low sample of patients with AVB. More recently, a publication from the Framingham cohort^4^ changed this paradigm. After 20 years of follow up, PR prolongation was associated with increased risk of atrial fibrillation, pacemaker implantation, and death^4^ . A large Danish ECG study with 288,181 patients confirmed the higher risk for atrial fibrillation associated with the presence of the first AVB.^24^

In our population, a 24% reduction in the survival rate of patients with PR > 200 ms, after adjustment for age, sex and clinical conditions were found, contrary to a previous study in the Finnish population.^25^ Some differences between these cohorts must be pointed out. The Brazilian cohort was older (mean age 51.7 versus 44 years), and it also included elderly patients. We analyzed about 1.5 million ECG versus 10,000. Chagas disease was relatively prevalent and it had a strong association with the presence of AVB, regardless of the degree. The social differences between both countries might also have contributed. Access to public health services and population education are completely unequal in low- and middle-income countries and may have a prognostic impact on the population.^26^

It is well established that irreversible Mobitz type II, high and third-degree AVB are indications for permanent pacing, even in asymptomatic patients.^3^ Their association with mortality is expected,^9^ since AV conduction injury is more severe, and heart disease is often related.^3^ The prognosis in 2:1 AVB is intimately related to the site of the AVB: nodal or infranodal.^3^ In the present study, 2:1 AVB in the 12-lead ECG was associated with a 79% reduction in relative survival, probably indicating an infranodal block. Mobitz type I AVB, on the other hand, was not associated with higher mortality in our cohort.

Mobitz type I AVB frequently has a benign prognosis, especially in young patients without cardiac disease.^27^ It can be a vagal mediated AVB that does not have an anatomical involvement of AV conduction,^28^ and it does not, therefore, progress to a high-degree AVB. In older patients, the natural history can be different, and they might benefit from a permanent pacemaker.^29^ We did not perform a sub-analysis in elderly patients, and the presence of symptoms is unknown.

Patients with cardiovascular emergencies often seek health assistance in primary care units, especially in small and remote counties. Tele-electrocardiography services play an important role in this setting, mainly for recognizing potentially life-threatening ECG abnormalities that are misdiagnosed by the local physician.^30^ In our service, second-degree AVB was statistically higher in the ECGs assigned as elective than in those with emergency priority.^30^ Patients’ outcomes could change with early referral to the hospital and consequent pacemaker implantation.^31^ Hospitalization data was not available for our entire cohort and, therefore, was not included in this paper. Nonetheless, further work in this field is planned to evaluate patients’ journey in our healthcare system from the ECG diagnosis of AVB.

### Limitations

Our study has limitations. The clinical data was self-reported and, thus, might have been underreported. The Lazy Associative Classifier software used to classify ECG reports has good accuracy, sensibility, and positive predictive value, but it may make errors. In order to minimize this problem, we included the Glasgow and Minnesota automatic classification in the diagnostic algorithm. Furthermore, manual revision of more than 9,000 ECGs was conducted to confirm the presence of AVB. The probabilistic linkage also has some issues, such as less than perfect sensitivity and the possibility of false pairs. Therefore, we defined a high cutoff point for true pairs and conducted manual revision for the doubtful cases. We still do not have information on symptoms or hospitalization data, but data from pacemaker procedures in each group will soon be available for analysis, and future work on this topic has been planned.

Nevertheless, our study brings new data on AVB prognosis, as it evaluates a Latinx population from a primary care centers with more than one million patients. Our findings are consistent and might be a useful tool to direct public health policies and funding resources.

## Conclusion

The presence of AVB was associated with an increased risk of overall mortality in the TNMG population. In patients with second and third-degree AVB, only those with Mobitz type I did not have a higher risk of mortality.
